# The Cause of Cholera

**Published:** 1850-03

**Authors:** 


### THE CAUSE OF CHOLERA.

All who are attentive observers of "the form and pressure of the times," have been struck with the facility with which the clergy cut the Gordian knots of medical philosophy. When physicians are puzzled, either about the cause or treatment of a disease, the clergy benevolently step in to clear up the mystery. The following statement, made by the London correspondent of the New York Tribune, is no fancy sketch, and it is illustrative of the remarks we have made on the medical tendencies of the clergy. The Tribune says:

"The cause of cholera not having been determined by physical examination, it is attempted to account for it upon spiritual reasons. Rev. Dr. McNeile thinks it is a judgment on England for favoring 'Popery'; Rev. Mr. Toye, of Gateshead, that it is to deter people from marrying deceased wives' sisters; Rev. Mr. Gutch, of Liecester, attributes it to Parliamentary electors voting for Dissenters and Jews, instead of Church of England men; while others attribute it to the omission of 'Dei Gratia,' from the new florin. The new Post Office Sunday arrangement is another assigned cause. An old Jew, being in an out-of-the-way alehouse, treated himself to some ham and eggs; but just as he was about to raise the forbidden morsel to his mouth, a clap of thunder startled him: he dropped his fork, saying 'Mein Gott! all dish fuss, chust becaush old Moshesh is eating a leedle pit of pacon!' We are constantly looking to the stars for the causes of things which lie at our feet. More than one educated man has told me in England that he believed Animal Magnetism to be a Satanic influence. A belief in present witchcraft, as a special compact with the old gentleman who wears the horns and flourishes the tail, was avowed to me by a classical scholar and extensive reader."

